# Virucidal and cytotoxic properties of a natural honeybee hives-derived formulation suspended with marine plasma

**DOI:** 10.3389/fcimb.2025.1666782

**Published:** 2025-11-11

**Authors:** Barbara Bażanów, Paweł Migdał, Aleksandra Chwirot, Agata Kublicka, Tomasz Gębarowski, Paweł Chorbiński, Andrzej Vogt, Antoni Szumny, Ewa Kaczmar, Katarzyna Michalczyk, Dominika Stygar

**Affiliations:** 1 Department of Pathology, Division of Microbiology, Faculty of Veterinary Medicine, Wrocław University of Environmental and Life Sciences, Wrocław, Poland; 2 Department of Bees Breeding, Institute of Animal Husbandry and Breeding, Wrocław University of Environmental and Life Sciences, Wrocław, Poland; 3 Department of Biostructure and Animal Physiology, Wrocław University of Environmental and Life Sciences, Wrocław, Poland; 4 Department of Epizootiology and Clinic of Birds and Exotic Animals, Faculty of Veterinary Medicine, Wroclaw University of Environmental and Life Sciences, Wrocław, Poland; 5 ZB-18a Magnetic Materials Group, University of Wrocław, Wrocław, Poland; 6 Department of Food Chemistry and Biocatalysis, Wrocław University of Environmental and Life Sciences, Wroclaw, Poland; 7 Department of Clinical Diagnostics, Faculty of Veterinary Medicine, University of Warmia and Mazury in Olsztyn, Olsztyn, Poland; 8 Department of Physiology, Faculty of Medical Sciences in Zabrze, Medical University of Silesia, Zabrze, Poland

**Keywords:** bee-derived products, cytotoxicity, honeybee cap extract, lysozyme, enhanced marine plasma, virucidal activity

## Abstract

**Introduction:**

Emerging viral pathogens continue to pose serious threats to global health, prompting the search for novel antiviral agents derived from natural sources. Bee-derived products, particularly those enriched with lysozyme, have shown promising antimicrobial properties. This study aimed to evaluate the virucidal and cytotoxic properties of a new formulation combining extract from bee brood caps (EBBC) and enhanced marine plasma as a potential broad-spectrum antiviral agent.

**Methods:**

The chemical composition of EBBC was characterized by gas chromatography–mass spectrometry (GC–MS) and lysozyme activity assays. Cytotoxicity was assessed using the sulforhodamine B (SRB) assay on normal epithelial (CCD841) and fibroblast (NHDF) cell lines. Virucidal activity was tested according to European (PN-EN 14476+A2:2019-08) and ISO (ISO 18184) standards. Additionally, the effect of EBBC on viral entry was analyzed using fluorescence microscopy.

**Results:**

EBBC demonstrated potent virucidal activity, achieving ≥99.99% efficacy at concentrations as low as 0.01% and maintaining full effectiveness after 12 months of storage. The formulation was active against all tested viruses. Cytotoxicity testing revealed minimal toxicity, with concentration-dependent inhibition of epithelial cell growth and stimulation of fibroblast proliferation.

**Discussion:**

EBBC exhibits strong and stable antiviral activity at low concentrations with minimal cytotoxic effects. Its unique combination of bee brood cap extract and enhanced marine plasma suggests potential for development as a natural, broad-spectrum antiviral formulation, offering an alternative to conventional antiviral agents.

## 1 Introduction

Despite promising to revolutionize human health and medicine (with potential breakthroughs in areas such as venom toxicology and allergic disease research), the Honeybee Genome Sequencing Project (HBGP) has resulted in limited progress, with therapeutic applications primarily confined to apitherapy (Hepburn et al., 1991). One of the major hurdles of bee-derived products is the non-specificity of therapeutic compounds. Bee-derived products include beeswax platelets, which are used to build the nest combs. The space between the combs is filled with air containing 56 different volatile compounds and microorganisms (Abd El-Wahed et al., 2021; Santorelli et al., 2023) or propolis produced to protect the hive against pests and coat the interior of the hive, most probably due to strong bactericidal and fungistatic properties (De Vecchi and Drago, 2007; Giampieri et al., 2022; Hegazi et al., 2000). The biological properties of propolis depend on its chemical composition, plant sources, geographical zone, and season. A total of 300+ compounds have been identified in propolis, including phenolic compounds, aromatic acids, essential oils, waxes, and amino acids (Anjum et al., 2019; Huang et al., 2014). These elements enable not only the survival of adults but also pupae and larvae, as the entire development cycle takes place in wax combs covered with propolis (Anjum et al., 2019; Huang et al., 2014). The brood is sealed with a wax cap on the tenth day after egg laying. The characteristics of honeybee caps play an important role in preventing the multiplication of bacteria and viruses, particularly within brood cells. The wax used to seal the cells consists of approximately 300 substances, including fatty acid esters, hydrocarbons, free fatty acids (Svečnjak et al., 2019; Tulloch, 1980), pollen grains, propolis, and threads from larval cocoons (Abou-Shaara, 2015; Abou-Shaara and Staron, 2018). The hydrophobic substances in the wax caps protect the larvae and honey within the cell against high humidity and yeast growth, and the caps apertures facilitate gas exchange, maintain optimal conditions within the brood cells, and permit the workers to monitor the health of the pupae (Kubásek et al., 2022; Mondet et al., 2021). As honeybees develop within these caps, their immune defense mechanisms—featuring compounds like defensins, prophenoloxidase (proPO), and lysozyme—are a growing area of investigation.

Lysozyme refers to a group of peptides with highly conserved chemical structures found across various life forms, including animals, plants, insects, bacteria, and even viruses (Bergamo and Sava, 2024; Callewaert and Michiels, 2010). Human lysozyme and the one derived from chicken egg white are the most well-known examples (Jollés and Jollés, 1984; Reitamo et al., 1978). It is noteworthy that chicken lysozyme is employed for therapeutic applications (Kiaerulff et al., 2018; Shteyngart, 2005). This enzyme possesses antimicrobial characteristics and may play a significant role in immunomodulation (Bergamo and Sava, 2024). Nevertheless, lysozyme is essential in the immune response mechanisms of the honeybee. The lysozyme found in honeybees is a low-molecular-weight basic protein, exhibiting a molecular mass of 13–17 kDa and displaying properties analogous to those of hen egg white lysozyme. It is classified as a true lysozyme of type C (chicken) (Jollès et al., 1979). The source of insect lysozyme is the fat body, which is the main organ of protein biosynthesis, including hemolymph proteins. Lysozyme has demonstrated efficacy in the management of chalkbrood disease within *Apis mellifera* colonies (Van Haga et al., 2012), which substantiates its antimicrobial properties.

Suspending bee-derived compounds with enhanced marine plasma, obtained from specific depths and environmental circumstances may result in the attainment of synergistic effects, particularly since deep-sea water demonstrates a harmful influence on certain viruses (influenza, herpes virus), bacteria (*Pseudomonas aeruginosa*, *Escherichia coli*), and hypertonic saline has been utilized in the prevention of influenza, bronchiolitis, rhinitis, sinusitis, and other respiratory disorders. The antiviral features of deep-sea water have been linked to metal ions, microalgae, microbes, and minerals (Ahmad et al., 2019; Kurhekar, 2020; Thorgersen et al., 2008; Tortorella et al., 2018). *In vitro* assays have demonstrated that hypertonic saline solution effectively prevents virus replication via membrane depolarization and intracellular energy deprivation, which is believed to inhibit the SARS-CoV-2 life cycle without affecting the hosts cell’s mitochondrial function (MaChado et al., 2021).

Considering the continuous threat posed by emerging viral infections to both humans and animals, the development of effective prevention and treatment strategies is critical. The presented study aimed to research the virucidal properties of EBBC, a novel combination of honeybee cap extracts and enhanced marine plasma, as a potential antiviral agent in the prevention of viral infections. The symbiosis of comparative genomics and hive inoculation represents a promising approach to the development of potent, specifically tailored, on-demand antimicrobial formulations, such as EBBC.

## 2 Materials and methods

### 2.1 Viruses for EBBC preparation

To create the EBBC preparation, inactivated viruses or virus mixtures were introduced into the hives. These viruses belonged to various families and differed in structure, type of nucleic acid, and the diseases they caused. They also varied in infection patterns, seasonality, and modes of transmission (**Table 1**). A total of 15 viruses were prepared, including human adenoviruses 3 (HAdV-3VR-847™, ATCC, USA), 4 (HAdV-4, VR-1572™, ATCC), 5 (HAdV-5, VR-5™, ATCC), 36 (HAdV-36, VR-1610™, ATCC), and 41 (HAdV-41, VR-930™, ATCC); vaccinia virus (VACV, VR-1536™, ATCC); herpes simplex virus (HSV-1, VR-1493™, ATCC); equine herpesvirus 1 (EHV-1, VR-700™, ATCC); poliovirus 1 (PV-1, LS-c 2ab, NIBSC); human rhinovirus 1a (HRV-1a, VR-1559™, ATCC); murine hepatitis virus 1 (MHV-1, VR-261™, ATCC); human coronavirus OC43 (HCoV OC43, VR-1558™, ATCC); murine norovirus (MNV, strain S99 Berlin, EVAg); influenza A virus (H1N1) (FLUAV, A/PR/8/34, VR-95™, ATCC); and equine arteritis virus (EAV, VR-796™, ATCC).

**Table 1:** Viruses used for extract from bee brood caps (EBBC) preparation.

Virus	Family	Size	Genetic material	Envelope
HAdV-3	*Adenoviridae* (subgroup B)	80 nm	dsDNA	–
HAdV-4	*Adenoviridae* (subgroup E)	80 nm	dsDNA	–
HAdV-5	*Adenoviridae* (subgroup C)	80 nm	dsDNA	–
HAdV-36	*Adenoviridae* (subgroup D)	80 nm	dsDNA	–
HAdV-41	*Adenoviridae* (subgroup F)	80 nm	dsDNA	–
VACV	*Poxviridae*	270 x 300 nm	dsDNA	+
HSV-1	*Herpesviridae*	100–120 nm	dsDNA	+
EHV-1	*Herpesviridae*	100–120 nm	dsDNA	+
PV	*Picornaviridae*	27–30 nm	ssRNA+	–
HRV	*Picornaviridae*	27–30 nm	ssRNA+	–
MHV-1	*Coronaviridae*	80–120 nm	ssRNA+	+
HCoV-OC43	*Coronaviridae*	80–120 nm	ssRNA+	+
MNV	*Caliciviridae*	23–40 nm	ssRNA+	–
FLUAV	*Orthomyxoviridae*	80–120 nm	ssRNA- segmented	+
EAV	*Arteriviridae*	45–60 nm	ssRNA+	+

EAV, equine arteritis virus; EHV-1, equine herpesvirus 1; FLUAV, influenza A virus (H1N1); HAdV, human adenovirus; HCoV, human coronavirus; HRV-1a, human rhinovirus 1a; HSV-1, herpes simplex virus 1; MHV-1, murine hepatitis virus 1; MNV, murine norovirus; PV-1, poliovirus 1; VACV, vacciniavirus.

Additionally, two virus sets were prepared: one comprising respiratory viruses (FLUAV, HRV-1a, HCoV OC43, and HAdV-5) and one comprising viruses causing eye infections (HSV-1 and HAdV-5).

#### 2.1.1 Preparation of viruses for hive inoculation

Virus suspensions were prepared using different cell lines: A549 cells (ATCC CRM-CCL-185™) for adenoviruses, Vero cells (ATCC CCL-81™) for poxviruses, HeLa cells (ATCC CRM-CCL-2™) for picornaviruses and herpesviruses, HT-29 cells (ATCC HTB-38™) for coronaviruses, 17Cl1 cells (RRID: CVCL_VT75) for caliciviruses, RK-13 cells (ATCC CCL-81™) for herpesviruses, and BHK-21 cells (ATCC CCL-10™) for arteriviruses. The cells were cultured in 175 ml flasks (NEST SCIENTIFIC Biotechnology, New Jersey, USA) containing Eagle’s Minimum Essential Medium (EMEM) with Earle’s Balanced Salt Solution (BSS) and 10% fetal bovine serum (FBS) (Capricorn Scientific, Germany). Virus suspensions were prepared from stock suspensions added to the cell monolayers and incubated for 2 h at 37 °C, with gentle shaking every 15 min. Once the cells displayed a cytopathic effect, they were subjected to three freeze-thaw cycles at -80 °C, followed by low-speed centrifugation (10 min at 1500 x g) to remove cell debris. The resulting virus suspensions were aliquoted and stored at -80 °C.

#### 2.1.2 Infectivity determination

Infectivity was determined using endpoint titration. For this, 0.1 ml of each virus dilution was added to 8 wells of a microtiter plate, starting from the highest concentration. Then, 0.1 ml of freshly trypsinized cells from the appropriate culture were added. The plates were incubated at 37 °C in a 5% CO_2_ atmosphere and observed daily for up to 7 days using an inverted microscope (Axio Observer, Carl Zeiss MicroImaging GmbH, Germany). The cytopathic effect was recorded, and the tissue culture infective dose (TCID_50_) required for subsequent stages of the study was calculated using the Spearman-Kärber method:


$$-\text{lo}{\text{g}}_{10}\text{TCI}{\text{D}}_{50}={\text{x}}_{0}–0.5+\text{Σ r}/\text{n}$$

where:

x_0_ – log10 of the lowest dilution with 100% positive reaction

r – number of positive determinations of lowest dilution step with 100% positive and all higher positive dilution steps

n – number of determinations for each dilution step

The replication of the influenza virus was determined using embryonated chicken eggs (ECEs). A 0.2 ml suspension of the influenza A/H1N1 strain A/PR/8/34 (H1N1) virus was inoculated into the allantoic cavity of ECEs. The ECEs were incubated at 35 °C for 3 days in an egg incubator. Then, the ECEs were cooled overnight in a refrigerator, and the allantoic fluid was collected. Hemagglutination assays were performed on the collected fluids to confirm the successful replication of the influenza virus. Before testing, the allantoic fluid containing the virus was diluted with phosphate-buffered saline (PBS) at pH 7.2 to achieve a virus concentration of 10^7^ EID_50_ (Egg Infective Dose at 50%) per 0.2 ml.

All propagated viruses, including the stock virus suspension, were inactivated using UV-C radiation (254 nm) for 30 min (Sterilon Flow 72W UV-C, Lena Lighting, Poland). Then, the inactivated virus suspensions were prepared for administration to the hives.

### 2.2 Producing and obtaining material for EBBC production

Material for EBBC production was obtained from the Carniolan honeybee colonies (*Apis mellifera carnica*, Pollmann) exposed to the selected viruses (2 bee colonies for each virus). For this process, 1 ml of the inactivated virus suspension was mixed with 49 ml of 1 mol/l sucrose syrup. This mixture was transferred to an atomizer and sprayed onto the bee colony (Koenig and Petersen, 2022) every 4 days for a total of 4 applications. The final spray was followed by an additional 4-day waiting period. Afterward, marked combs were swept from the bees, and caps were collected in the apiary laboratory using a sharp, sterile knife, minimizing brood damage. In addition, caps were taken from a hive not inoculated with any viruses. The collected caps were placed in sterile string bags and immediately stored in a freezer at -20 °C for further processing. The complete experiment was carried out in a research apiary at Wrocław University of Environmental and Life Sciences; following the exposure, the bee colonies were prepared for wintering.

### 2.3 Procedure for extracting material from caps

To obtain the extract from caps, 330 mL of sterile ddH_2_O was added to a mixer (Tefal, Rumilly, Haute-Savoie France; Philips, Amsterdam, Netherlands) operating at 30,000-35,000 rpm. Then, small portions of raw material (about 20 g of each cap) were added gradually, and the mixture was stirred for 5 min. The resulting cloudy, beige-white suspension was then transferred to a bucket centrifuge (MPV MedInstrument, Warsaw, Poland) and centrifuged for 10 min at 10,000 rpm. The clear supernatant was transferred to a glass buffer container. The remaining suspended precipitate, primarily waxes, was gradually added (every 5–8 s) to a stirrer containing 160 mL of sterile, distilled, and demineralized water. The mixture was stirred for 5 min at 30,000 rpm and then centrifuged for 5 min at 10,000 rpm. The resulting supernatant was transferred to the buffer container. The liquid was filtered using Schott glass sieves type G-3 or G-4 (Chemland, Stargard, Poland) or 0.45 μm membrane filters (Advantec, Tokyo, Japan) to ensure clarity. The filtered liquid, now ranging in color from pale yellow to brown, was transferred to a vacuum rotary evaporator (Heidolph, Schwabach, Bayern, Germany). The water was evaporated at 38-41 °C under a vacuum of 0.15-0.20 bar, with the evaporator rotating at 180–200 rpm. To extract the final product from the evaporator flask, 30 mLof sterile water was added, and the product was dissolved by rotating the flask. This concentrated EBBC solution was transferred to a conical flask. The flask was rinsed several times to ensure complete product transfer, and all solutions were combined. To remove any residual water, the final solution was heated in a water bath (Heidolph Scientific Products, Schwabach, Germany) at 36-38 °C or under infrared heaters (MSW Motor Technics, Berlin, Germany) while a stream of nitrogen gas (N_2_) was introduced through specially designed gas nozzles. The containers were placed in a thermostatic shaker (Electrothermal™ STEM RS1000, Fisher Scientific Co LLC, Pittsburgh, PA, USA) for about 2 h. Once complete evaporation was achieved, the containers were sealed while the nitrogen stream continued and stored in a refrigerator at 4 °C. Final extraction yield ranged between 0.5-1.0 g.

### 2.4 Determination of EBBC composition

#### 2.4.1 Chemical composition studies

The chemical profile of EBBC from hive inoculated by HSV-1 was evaluated using a N,O-bis(trimethylsilyl)trifluoroacetamide (BSTFA) derivatization method, following the protocol of Kuropka et al (Kuropka et al., 2021; Pachura et al., 2022), and analyzed by GC-MS (Shimadzu QP 2020, Shimadzu, Kyoto, Japan) was used for identification of the constituents. In brief, 500 µL of pyridine and 50µL of BSTFA were added to the freeze-dried sample of EBBC (approximately 20 mg). Next, the mixture was transferred to a vial and heated for 40 min at 60 °C. The separation was achieved using a Zebron ZB-5 capillary column (30 m, 0.25 mm, 0.25 μm; Phenomenex, Torrance, CA, USA). GC-MS analysis was performed according to the following parameters: scans were performed from 40 to 1050 m/z in electron impact ionization (EI) at 70 eV with a mode of 10 scans per second. Analyses were performed using helium as a carrier gas at a flow rate of 1.0 mL/min at split 1:20, and the following program: 100 °C for 1 min, 2.0 °C/min temperature rise from 100 to 190 °C; 5 °C/min temperature rise from 190 to 300 °C. The injector was maintained at 280 °C, respectively. Compounds were identified by comparing both their retention times and mass spectra to known standards, to enable comparison. Retention times were matched against a standard mixture of saturated alkanes (C7-C40) from Centrum Odczynników Chemicznych CRCH sp. z o.o., while mass spectra were compared to a library of known compounds (Willey NIST 23). A match was considered successful if the fit index was greater than 90%. Two programs: AMDIS (v. 2.73) and GCMS solution (v. 4.20) Shimadzu Lab Japan, were used to process the spectra. Integration of chromatograms files was performed by ACD Spectrus Processor v. 2021.2.1 (Advanced Chemistry Development, Inc. Toronto, Ontario Canada).

#### 2.4.2 Testing for lysozyme presence and activity

Four different types of EBBC samples were selected for lysozyme activity analysis:

1. From a non-inoculated hive.
2. Non-filtered from the hive inoculated with HCoV-OC43.
3. Filtered from the hive inoculated with HCoV-OC43.
4. From the hive inoculated with HSV-1.

A dilution series was performed for each sample to create final concentrations of 1%, 0.5%, and 0.25%. Two technical replicates were prepared for each concentration. To establish the background lysozyme activity, control measurements were conducted using the enhanced marine plasma-diluent.

The lysozyme activity was measured using the EnzChek Lysozyme Assay Kit (catalog number: E22013, Thermo Fisher Scientific, Waltham, MA, USA) following the manufacturer’s instructions. The assay results were read using a Synergy H1 multiplate reader (Agilent Technologies, Santa Clara, CA, USA) set to fluorescence mode (with excitation at 490 nm and emission at 520 nm).

### 2.5 EBBC formulation

Isotonic enhanced marine plasma was added to the prepared extracts to create a 10% clear solution, serving as the base formulation of EBBC for testing. Some of the formulations were filtered using a 0.22 µm Millex-GS syringe filter (Merck Milipore, Burlington, MA, USA).

### 2.6 EBBC cytotoxicity studies

#### 2.6.1 Cell cultures

Cytotoxicity studies were conducted using standard protocols with cell lines sourced from the cell culture laboratory’s biobank. The cells were cultured for a minimum of two weeks prior to the experiment. The following cell lines were used: CCD 841 CoTr (colorectal epithelioma, CRL-1807™, ATCC, USA) and NHDF (normal human dermal fibroblasts, CC-2511, LONZA, Belgium). Each line was maintained in specific media: EMEM for CCD 841 CoTr and DMEM for NHDF, supplemented with fetal bovine serum (FBS), L-glutamine, and antibiotics. The cells were cultured at 37 °C in a humidified atmosphere with 5% CO_2_.

#### 2.6.2 SRB test

For evaluating the biological activity of EBBC, the SRB assay, which is recommended for assessing cytotoxicity and cell growth, was utilized. This assay measures these parameters by determining the total protein content in the cells.

In the screening experiment, cells were inoculated into 96-well multi-well plates in 200 μl at a density of 20,000 cells/well. After cell inoculation, plates were incubated at 37 °C, 5% CO_2_, for 24 h before adding other EBBC. Then, Trichloroacetic acid (TCA) was fixed from each cell line to represent the cell population measurement for each cell line at the time of compound addition (Tz). At the same time, EBBC samples were added to the cells at 5 concentrations (0.5%, 1%, 2.5%, 5%, 10%). Next, the plates were incubated for an additional 48 h. After the incubation, the cells were fixed with 50 μl of cold 50% (w/v) TCA (final concentration, 10% TCA) and incubated for 60 min at 4 °C. The supernatant was discarded, the plates were washed five times with water, and air-dried. Then, 100μl of 0.4% (w/v) sulforhodamine B (SRB) solution in 1% acetic acid was added to each well, and the plates were incubated for 30 min at room temperature. After staining, the unbound dye was removed by washing the plates five times with 1% acetic acid, and then they were air-dried. The bound dye was then dissolved with 10 mM trizma base, and the absorbance was read on an automated plate reader at 515 nm (MultiskanGO, Thermo Fisher Scientific, Waltham, MA, USA) for zero time (Tz), control growth (C), and test growth in the presence of the EBBC at five concentration levels (Ti). Percentage growth was calculated as:


[(Ti−Tz)/(C−Tz)] × 100 (for concentrations for which Ti≥Tz)

Or


[(Ti−Tz)/(C−Tz)] × 100 (for concentrations for which Ti<Tz)

### 2.7 *In vitro* testing of the virucidal properties of EBBC

The presented study determined the virucidal efficacy and the lowest effective concentration of the product, the shelf life of the product, the specificity of virucidal activity, and the shortest contact time (the amount of time a pathogen may be in contact with a disinfectant).

#### 2.7.1 Virucidal activity according to European Standard PN- EN 14476+ A2:2019-08

Virucidal activity for different concentrations of EBBC (ranging from 0.001% to 10%) and various contact times (5, 10, 15, and 60 min) were tested. Virucidal activity of EBBC against viruses not originally inoculated into the hive was also evaluated.

For more convenient handling, the volumes used in the assay were: 0.1 ml test virus suspension, 0.1 ml interfering substance (PBS) and 0.8 ml EBBC (in different concentrations 0,0001%, 0,001%, 0,01%, 0,1%, 1%, 2%, 5%, 10%). The product’s activity was stopped by dilution to 10–^12^ immediately at the end of the contact time. Each dilution (10^-1^, 10^-2^, 10^-3^,10^-12^) in 8 repetitions and cell cultures appropriate to the tested viruses was added onto 96-well polystyrene plates (Thermofisher Scientific) which were then placed in an incubator (Eppendorf, Hamburg, Germany) at 37 °C, 5%CO_2_ for 3–7 days, depending on the virus tested. In parallel, the titer of the control virus (not treated with EBBC) was tested. After the incubation period, the titers of the EBBC-treated virus and the control virus were read, compared, and the degree of reduction was estimated using the Spearman-Kärber method.

According to the European standard: PN- EN 14476+ A2:2019-08, a preparation is considered virucidal if, after the recommended exposure time, the virus titer is reduced by at least 4 log10 (inactivation ≥ 99. 99%).

#### 2.7.2 Virucidal activity according to ISO 18184 standard

Influenza virus testing, following ISO 18184 guidelines, used embryonated chicken eggs (ECEs) as the testing substrate. The study utilized 10-day-old chicken embryos from Rossa hens.

The allantoic cavity of ECEs was inoculated with a 0.2 ml suspension of FLUAV H1N1 (HA titer of 64) with 0.1% EBBC (n = 3) or a 0.2 ml suspension of control virus (of the same hemagglutination unit) (n = 3). The inoculated ECEs were incubated for two days at 35°C. After the incubation period, the allantoic fluid from the eggs was collected and two-fold serial dilutions were prepared. Next, a 0.5% suspension of chicken red blood cells was added to each dilution. The tubes were observed for hemagglutination, which would indicate virus replication. The EID_50_ (Egg Infectious Dose 50%) titer of the virus was then calculated using the Reed and Muench method, based on the cumulative number of infected embryos. Antiviral efficacy (Mv) was calculated using the following formula:


Mv=−lg(Vb/Va)=−[lg (Vb)−lg(Va)]

where:

Mv is the antiviral efficacy value

lg (Vb) represents the EID_50_ of infected embryonic allantoic fluid without contact with the test sample after a defined contact time

lg(Va) represents the EID_50_ of infected allantoic fluid from embryos in contact with the sample after a defined contact time.

According to the ISO 18184 standard, a preparation is considered virucidal if, after the recommended exposure time, the virus titer is reduced in comparison with the control virus. EBBC was tested against influenza virus A/H1N1, at a concentration of 0.1%. The exposure time was 60 minutes. After this time, EBBC was inoculated into ECEs and (after incubation period), a titer reduction was determined using hemagglutination assay.

#### 2.7.3 The shelf life of the product

The virucidal efficacy of the product was tested according to the above standards immediately after preparation and after 12 months of storage.

#### 2.7.4 The specificity of virucidal activity of EBBC

The specificity of virucidal activity of different types of EBBC was tested according to European Standard PN- EN 14476+ A2:2019–08 against: MHV-1, HSV-1, HAdV-C5.

#### 2.7.5 The shortest contact time of the EBBC action

Virucidal activity of EBBC was tested according to European Standard PN- EN 14476+ A2:2019–08 at various time points: 5, 10,15 and 60 minutes.

### 2.8 Testing inhibition properties of EBBC against virus entry into cells by endocytosis

The Vero cell line and equine arteritis virus (EAV) were used to investigate the mechanism of action of EBBC as an inhibitor of viral entry *via* endocytosis, with ammonium chloride (NH_4_Cl) as a positive control (inhibitor). NH_4_Cl was dissolved in EMEM culture medium and filtered through a 0.22 µm syringe filter. EBBC was diluted in enhanced marine plasma. Vero cells were seeded on glass coverslips at a density of 3 × 10^5^ cells per ml in a 24-well plate. EBBC and NH_4_Cl were diluted in cell culture medium to obtain 1%- and 25-mM solutions, respectively. The cells were then pre-incubated with the tested substances for 2 h at 37 °C. After this time, the substances were removed and the virus (MOI 50 -multiplicity of infection) was added. The cells were incubated at 4 °C for 1 h to synchronize virions on the cell surface. Unbound virus was washed off with PBS and EMEM medium supplemented with 2% FBS. Then EBBC and NH_4_Cl at the indicated concentrations were added. Virus entry was initiated by returning the cells to 37 °C and the process was analyzed 3 h after infection. For this purpose, the cells were fixed with 4% paraformaldehyde (ThermoFisher Scientific, Waltham, MA, USA), permeabilized with 0.3% Triton X-100 (BioShop, Burlington, ON, Canada) and blocked with 3% BSA (Albumin Bovine Fraction, BioShop, Burlington, ON, Canada) for immunostaining. Each step was followed by three washes with PBS. Fixed cells were subjected to immunostaining with the following reagents: primary antibody, a monoclonal anti-N virus nucleocapsid protein antibody (Equine Arteritis Virus EAV MAb IgG1 Isotype, VMRD, Pullman, WA, USA); secondary antibody, goat anti-mouse IgG Alexa Fluor^®^ 488 (Abcam, Cambridge, UK); phalloidin-iFluor 594 reagent conjugate (Abcam, Cambridge, UK) for actin filaments; and DAPI reagent (Sigma Aldrich, Burlington, MA, USA) for nuclear staining. Fluorescence microscopy was performed using an inverted light microscope (Axio Observer) with a Zeiss 100x A-Plan objective.

## 3 Results

### 3.1 GC-MS analysis

**Figure 1:**
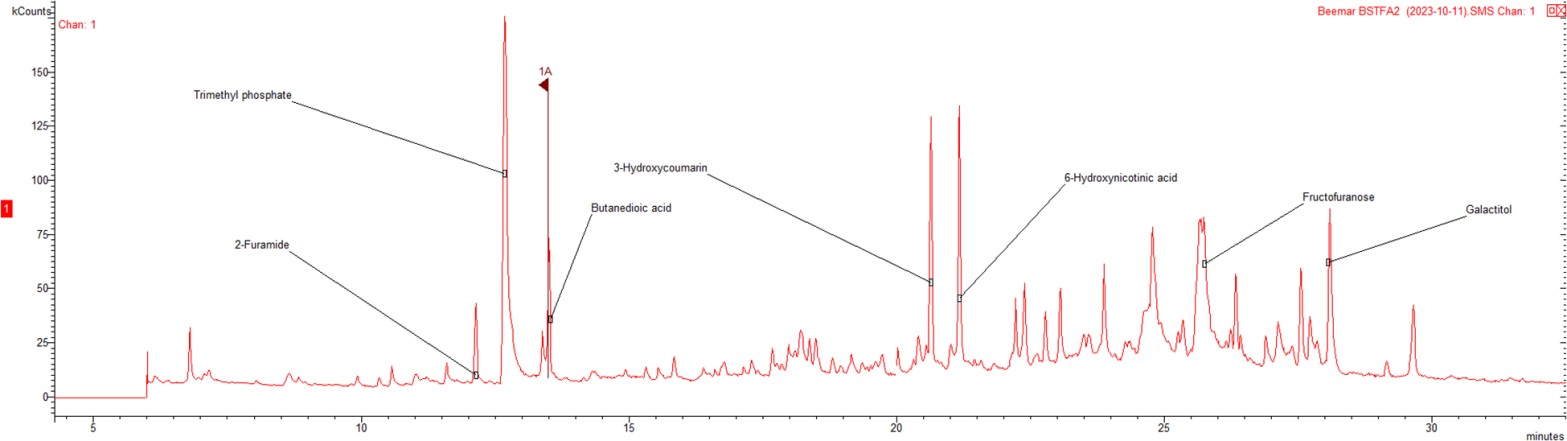
Sample GC-MS chromatogram of extract from bee brood caps (EBBC).

The GC-MS profile of EBBC reveals the presence of 36 compounds with a predominated carbohydrate group (**Figure 1**, **Table 2**). In addition, low- and medium-molecular acids such as benzoic, butanedioic, and entanedioic were identified. The hydroxy derivatives of coumarin, salicylic acid and resorcinol are among the compounds that could have the strongest antiviral potential (Pal et al., 2023; Singh et al., 2004).

**Table 2:** Chemical composition (GC-MS) of BSFTA derivatized components of extract from bee brood caps (EBBC).

Peak no.	Peak name*	tR (min)	Area (%)
1	Lactic Acid	6.799	1.779
2	Glycolic acid	7.160	0.214
3	Levoglucosenone	8.048	0.183
4	3-Pyridinol	8.662	0.603
5	3-Hydroxy-2,3-dihydromaltol	8.829	0.282
6	4-Hydroxy-5-methylfuran-3(2H)-one	9.921	0.362
7	Glycerol	10.340	0.299
8	Maltol	10.576	0.720
9	5-Hydroxymethylfurfural	11.021	0.363
10	Benzoic Acid	11.593	0.708
11	2-Furamide	12.142	2.472
12	Trimethyl phosphate	12.676	20.548
13	2-Furancarboxaldehyde	13.388	0.972
14	Butanedioic acid	13.503	3.250
15	Resorcinol + Fumaric acid	14.329	1.065
16	Pentanedioic acid	15.848	0.707
17	Elemicin	16.390	0.677
18	1-Dodecanol	16.784	0.697
19	D-Erythro-Pentopyranose, 2-deoxy-	18.485	1.000
20	4-Hydroxycoumarin	19.738	1.502
21	3-Hydroxycoumarin	20.644	6.745
22	6-Hydroxynicotinic acid	21.178	7.044
23	mono-Methyl terephthalate derivative	22.227	2.012
24	m- Salicylic acid	22.393	2.392
25	cis-1,4-Cyclohexanedicarboxylic acid,	23.869	3.545
26	Benzoic acid, 2-heptyl-6-hydroxy-	24.782	4.206
27	β-D-Tagatopyranose, 1,2,3,4,5-pentakis-O-TMS	25.342	0.865
28	β-D-Fructofuranose	25.673	16.851
29	Glucose	26.328	1.961
30	Glucose anomer	27.541	3.209
31	(3-Amino-1-benzofuran-2-yl)(phenyl)methanone	27.719	1.302
32	Galactitol,	28.091	6.566
33	Mannopyranose	29.157	0.602
34	Palmitic Acid	29.651	3.115
35	Oleic Acid	32.740	0.686
36	Stearic acid	33.200	0.498

N,O-Bis(trimethylsilyl)trifluoroacetamide, *Expressed as trimethylsilyl derivatives.

### 3.2 Lysozyme concentration and activity

The analyses confirmed that all tested samples contained lysozyme (**Table 3**). The results indicate increased lysozyme production in hives inoculated with viruses. In addition, the results show that filtrating the samples yielded higher lysozyme activity in the test preparations. Due to the characteristics of the test material and background, the study could only be conducted on highly diluted material. This results in a higher than standard test error, as it was performed at the limit of sensitivity of the test used.

**Table 3:** Lysozyme concentration (U/ml) in inoculated with filtered or non-filtered EBBC anti -human coronavirus OC43(CoV OC43) or anti-herpes simplex virus type 1 (HSV-1) viruses.

EBBC sample	Lysozyme activity (U/ml)
Non-inoculated	4.52 ± 0.94
Inoculated with HCoV-OC43 filtered	8.19 ± 0.49
Inoculated with HCoV-OC43 non-filtered	7.39 ± 0.25
Inoculated with HSV-1	6.93 ± 0.47

EBBC, extract from bee brood caps; HCoV, human coronavirus; HSV-1, herpes simplex virus 1.

Results are presented as mean ± SD.

### 3.3 EBBC cytotoxicity tested by SRB method

**Figure 2:**
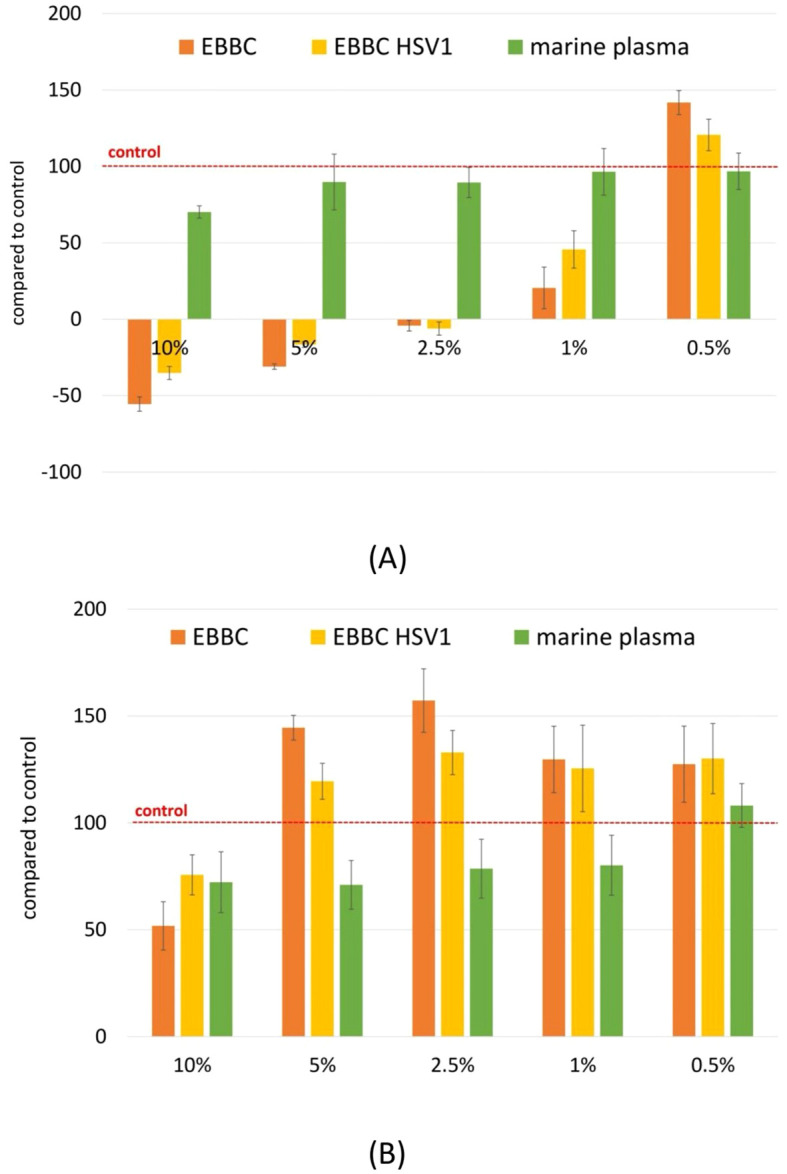
Evaluation of biological activity of EBBC in SRB assay for normal epithelial cells CCD841 **(A)** and fibroblasts NHDF **(B)**. Scores below 0 indicate cytotoxicity, scores ranging from 0 to 100 indicate inhibition of cell growth, scores above 100 indicate stimulation of proliferation. Abbrevations: HSV-1– herpes simplex virus 1.

The results show that the cytotoxic effect varies depending on the type of cells and their growth rate. In the case of normal epithelial cells (CCD841), we observed a strong inhibition of growth, whereas fibroblasts (NHDF) exhibited increased proliferation. The obtained results were concentration-dependent (**Figure 2**). Inhibition of epithelial cell growth may have a beneficial chemopreventive effect, since their excessive proliferation is key to cancer development. An increase in the number of fibroblasts, cells that proliferate slowly, indicates the ability of the compounds to stimulate regenerative processes.

### 3.4 Virological tests

#### 3.4.1 Research according to the European standard: PN- EN 14476+ A2:2019-08

##### 3.4.1.1 The lowest effective concentration

EBBC was effective even at 0.01% concentration, and at 0.001% concentration EBBC reduced the virus titer by 2 log (virucidal efficacy of 99%) (**Table 4**). Nevertheless, the most reliable concentration of EBBC at which we observed 99.99% efficacy against all viruses tested was 0.2%.

**Table 4:** Comparison of virucidal properties of different concentrations of extract from bee brood caps (EBBC) against herpes simplex virus 1 (HSV-1).

EBBC concentration	Virus reduction (log)	Virus reduction (%)
10%	4 log 10	99.99
2%	4 log 10	99.99
1%	4 log 10	99.99
0.5%	4 log 10	99.99
0.25%	4 log 10	99.99
0.2%	4 log 10	99.99
0.1%	3.875 log 10	99.987
0.08%	4 log 10	99.99
0.05%	3 log 10	99.9
0.01%	4 log 10	99.99
0.005%	3 log 10	99.9
0.001%	2 log 10	99

The virucidal efficacy of EBBC complex tested according to the European standard: PN- EN 14476+ A2:2019–08 at 0.2% concentration against various viruses is shown in **Table 5**.

**Table 5:** The virucidal efficacy of EBBC complex was tested according to the European standard: PN - EN 14476+ A2:2019–08 at 0.2% concentration against various viruses.

Tested virus	EBBC specificity	Virucidal activity
HAdV-3	Anti HAdV-3	5 log
HAdV-4	Anti HAdV-4	4
HAdV-5	Anti HAdV-5	4
HAdV-36	Anti HAdV-36	4
HAdV-41	Anti HAdV-41	4
VACV	Anti VACV	5
HSV-1	Anti HSV-1	4
EHV-1	Anti EHV-1	> 5
PV	Anti PV-1	5
HRV	Anti HRV	4
MHV-1	Anti MHV-1	4
HCoV-OC43	Anti HCoV-OC43	4
MNV	Anti MNV	4
EAV	Anti EAV	4

EAV, equine arteritis virus; EBBC, extract from bee brood caps; EHV-1, equine herpesvirus 1; FLUAV, influenza A virus (H1N1); HAdV, human adenovirus; HCoV, human coronavirus; HRV-1a, human rhinovirus 1a; HSV-1, herpes simplex virus 1; MHV-1, murine hepatitis virus 1; MNV, murine norovirus; PV-1, poliovirus 1; VACV, vaccinia virus.

##### 3.4.1.2 The length of shelf life

**Figure 3:**
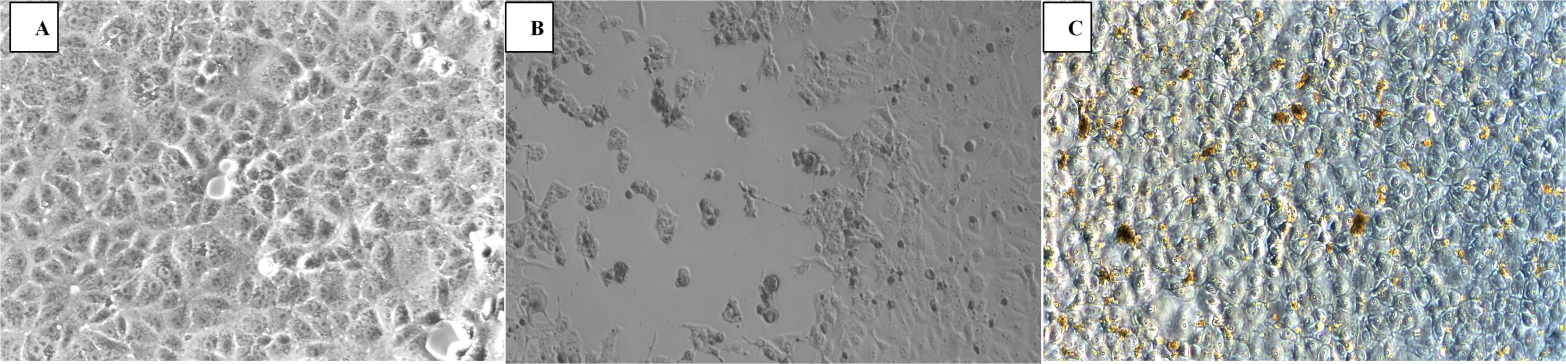
Antiviral potential of the sample EBBC against vaccinia virus after one year (the length of shelf life): **(A)** Control – uninfected Vero cells, **(B)** VACV infected Vero cells (positive control). **(C)** 1% EBBC + VACV infected Vero cells. No visible cytopathic effect. Mag.100x.

The virucidal efficacy EBBC after one year has not changed (99.99%) (**Figure 3**).

##### 3.4.1.3 The specificity of virucidal activity

**Figure 4:**
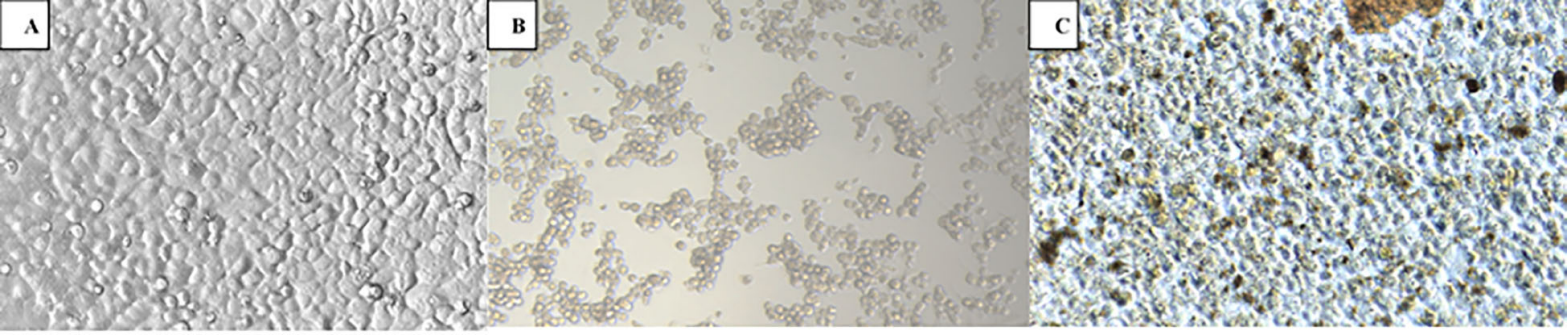
Antiviral potential of EBBC sample against human adenovirus type 5 used against HAdV-D36 (the specificity of virucidal activity): **(A)** Control – uninfected A549 cells, **(B)** HAdV-C5 infected A549 cells (positive control). **(C)** 1% EBBC + HAdV-C5 infected A549 cells. No visible cytopathic effect. Mag. 100x.

EBBC showing virucidal activity against a particular virus also worked against other viruses (effectiveness varied between 99.90% and 99.99%) (**Figure 4**).

##### 3.4.1.4 The shortest contact time

0.2% EBBC presented virucidal activity at the desired level at all contact times tested: 5, 10, 15, and 60 min.

#### 3.4.2 Virucidal efficacy against influenza virus- research according to ISO 18184 standard

The analysis (**Table 6**) of virucidal properties of 0.1% EBBC against influenza virus A/H1N1 in the embryonated chicken eggs (ECEs) showed that the antiviral efficacy (Mv) was 6 logs.

**Table 6:** Virucidal properties of 0.1% EBBC against influenza virus A/H1N1 were tested according to the ISO 18184 standard using the embryonated chicken eggs (ECEs).

Type of inoculation to allantoic fluid of ECEs	Dilutions									
	1:2	1:4	1:8	1:16	1:32	1:64	1:128	1:256	1:516	1:1032
0.1% EBBC + A/H1N1virus (n = 3)	–	–	–	–	–	–	–	–	–	–
	–	–	–	–	–	–	–	–	–	–
	–	–	–	–	–	–	–	–	–	–
A/H1N1 virus (n = 3)	+	+	+	+	+	+	–	–	–	–
	+	+	+	+	+	+	–	–	–	–
	+	+	+	+	+	+	–	–	–	–

- Precipitation of red blood cells is observed, but without condensation, + condensation of red blood cells is observed. EBBC, extract from bee brood caps.

### 3.5 Inhibition of virus entry into the cell via endocytosis

**Figure 5:**
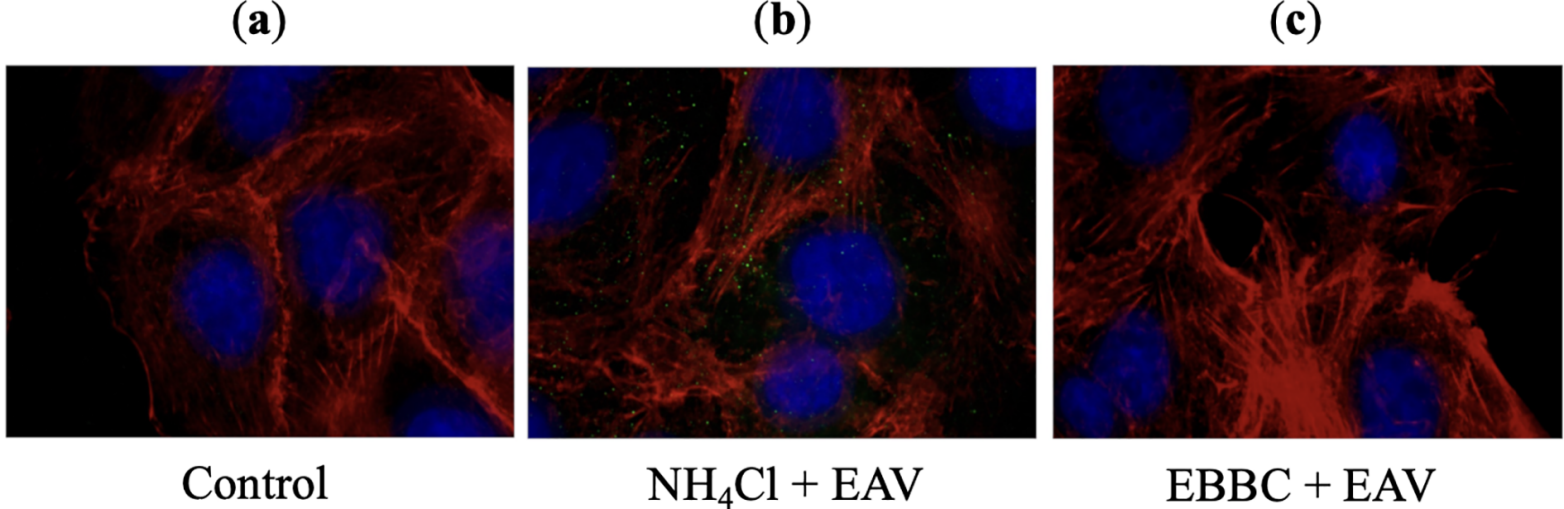
Effects of NH_4_Cl (inhibitor) and EBBC on equine arteritis virus (EAV) virions uncoating process 3 h after infecting Vero cells: **(A)** Control – uninfected Vero cells, **(B)** 25 mM NH_4_Cl (inhibitor) + EAV infected Vero cells. The fluorescence signal in the form of green dots corresponds to virions trapped inside endosomes, **(C)** 1% EBBC + EAV infected Vero cells. There is no virus-specific fluorescence signal.

Fluorescence microscopy utilized to visualize the process of viral entry into host cells, allowed the observation of key stages such as attachment, internalization, and uncoating. In cells untreated with inhibitor (**Figure 5A**), as well as those treated with EBBC (**Figure 5C**), the fluorescence signal gradually diminished post-uncoating, as the labeled proteins were released and subsequently dispersed or degraded. This uncoating occurred at the fusion site between the endosomal membrane and the viral envelope, approximately 3 h post-infection in our experiment. In contrast, cells treated with the active inhibitor NH_4_Cl (**Figure 5B**) retained the fluorescence signal at the same time point, reflecting the inhibition of viral release from the endosome. Our investigation into EBBC’s effect on the EAV entry process demonstrated that EBBC does not inhibit the viral uncoating stage, as was observed with NH_4_Cl.

## 4 Discussion

EBBC is a natural-based product combining substances used by bees to protect their brood and carried in an enhanced marine plasma solution, containing up to 78 bioavailable electrolytes and trace elements. GC-MS analysis of the investigated mixture identified 36 compounds with carbohydrates being the predominant group. However, as Kar et al. (2023) point out, simple carbohydrates generally lack the ability to inhibit viral growth or replication. Among the other compounds detected were organic acids, such as lactic, fumaric, butanedioic (succinic), and benzoic acids, all known for their significant antimicrobial properties. According to Wang et al. (2022), lactic acid, a metabolite produced by Lactobacilli, exhibits antiviral activity through physical binding to viruses, leading to envelope disruption and virion lysis, especially in enveloped viruses. Lactic acid has also been reported as a potential inhibitor of HIV replication in cases of altered genital bacterial flora in sub-Saharan African women (Gosmann et al., 2017), and has shown inhibitory effects on herpes simplex virus (HSV) replication (Eguchi et al., 2019), although its precise mechanism remains unclear. Another key compound identified, butanedioic acid (succinic acid), is known to act as a natural suppressor of antiviral immune responses by targeting mitochondrial antiviral signaling (MAVS) (Xiao et al., 2022). Xiao et al. (2022) discovered that butanedioic acid inhibits the MAVS-TBK1-IRF3 signaling pathway by preventing MAVS aggregate formation. Succinic acid also impairs influenza virus infection by succinylating and retaining the viral nucleoprotein within the nucleus, altering its interactions with viral RNA and disrupting the trafficking of viral ribonucleoprotein complexes (Guillon et al., 2022). Additionally, fumaric acid, in its dimethyl derivative form, has been shown to induce a cellular antiviral response that significantly inhibits SARS-CoV-2 replication across different cell lines (Olagnier et al., 2020). Moreover, aromatic hydroxycoumarins have been identified *via* molecular docking as potential anti-COVID-19 agents (Ait-Ramdane-Terbouche et al., 2020; Abdelmohsen et al., 2021)

What seems particularly significant, however, is that EBBC has been shown to contain lysozyme at concentrations ranging from 4.52 to 8.19 U/ml which contributes to its antiviral efficacy. The studies conducted on EBBC have demonstrated significantly higher levels of lysozyme in preparations derived from inoculated hives compared to those from uninoculated hives. This increase in lysozyme levels is likely attributed to the stress induced by bee spraying during hive inoculation. In bees, stress responses are known to trigger the upregulation of various immune-related compounds, including lysozyme, as part of their defense mechanisms. When bees experience stress, whether from environmental factors or external interventions like spraying, their immune systems become more active, leading to an increased production of antimicrobial peptides such as lysozyme (Lazarov et al., 2016). This response is similar to that observed in other organisms, where stressors, including pathogen exposure, enhance immune activity to protect the organism from potential infections. The elevated lysozyme levels in EBBC system from inoculated hives could contribute to its enhanced antimicrobial and antiviral activity. Lysozyme plays a key role in innate immunity by breaking down bacterial cell walls and interacting with viral particles, thereby providing a stronger protective effect in these preparations. Consequently, preparations from inoculated hives may exhibit superior therapeutic potential due to their higher lysozyme content, enhancing the overall efficacy of the formulation in combating viral and bacterial infections. This observation underscores the importance of environmental factors and hive conditions in influencing the bioactive properties of bee-derived products, opening avenues for further exploration of stress-induced immune responses in bees and their applications in antiviral therapies. The composition of EBBC is complex, and with such a wide variety of agents, it is reasonable to assume that its virucidal mechanisms may be multiple. However, due to the significant presence of lysozyme, as proved by the presented research, this substance seems to play a key role in the antiviral effect. Although lysozyme’s antiviral properties are less well understood, research dating back to the late 1950s reported its efficacy against herpes simplex, herpes zoster, warts, condylomata acuminata, aphthosis, and vaccinia viruses (Ferrari et al., 1959). Studies have shown that lysozyme levels in herpes zoster patients are lower in the infected eye compared to the healthy eye, suggesting a link between lysozyme and viral resistance (Eylan et al., 1977; Saari et al., 1983). This observation supports the potential application of EBBC as an eye drop for treating viral eye infections. In insects, lysozyme has been linked to antiviral immunity. For example, viral infections in silkworms (*Bombyx mori*) caused overexpression of lysozyme C (BmC-LZM), which significantly inhibited virus replication and increased larval survival during the late stages of infection (Chen et al., 2018). Although the mechanism of lysozyme from honeybees has not yet been fully described, studies on human and chicken lysozyme suggest that it can bind to nucleic acids, such as DNA and RNA (Steinrauf et al., 1999; Zalar et al., 2023). Furthermore, it has been shown to participate in viral transcription and replication, as demonstrated in studies on HIV (Brunaugh et al., 2021; Lee-Huang et al., 1999). The antiviral activity of lysozyme is thought to arise from its ability to interact with nucleic acids due to its positive charge, which alters the charge and electrophoretic mobility of DNA. This can disrupt vital processes such as transcription, translation, and cell division, thereby inhibiting viral replication. Lysozyme also contains DNA-binding motifs at its N- and C-terminals and can control RNA polymerase activity. These mechanisms may collectively limit virus-induced infections (Bergamo and Sava, 2024). This exposure of hydrophobic amino acids may also explain the enhanced antiviral activity, as these residues can interact with viral structural proteins, leading to viral inactivation (Ibrahim et al., 1996). For example, heat-denatured lysozyme has been shown to counteract Foot and Mouth Disease Virus (FMDV) by destroying capsid proteins and reducing viral titer by 2.7 log (Fukai et al., 2021). In contrast, EBBC, a non-heat-treated formulation, demonstrated even greater virucidal activity, achieving reductions in viral titers of at least 4 log (99.99% efficacy). EBBC ‘s broad-spectrum virucidal activity is promising for the prevention of respiratory infections, particularly given lysozyme’s established efficacy against various strains of influenza A viruses and even avian flu viruses like H5N1, H5N6, and H7N1 (Bergamo and Sava, 2024; Huang et al., 2023). The hypothesis that influenza viruses decrease lysozyme levels in the respiratory tract, possibly as a strategy to increase their infectivity, adds another layer of relevance to EBBC in respiratory care (Yamamoto and Yamamoto, 2022). Additionally, lysozyme has been found to deactivate coronaviruses such as MERS-CoV and SARS-CoV-2 by disrupting their viral envelopes, effectively blocking entry into host cells. This has led to lysozyme being included among antimicrobial peptides with potential applications in COVID-19 therapy (Brunaugh et al., 2021; Saini et al., 2022). Lysozyme’s specific activity varies across different sources but is highly potent, even at ng/ml concentration levels (Huang et al., 2023).

EBBC complex demonstrates exceptional virucidal efficacy (≥ 99.99%) with low toxicity, presenting, by offering high efficacy and user’s comfort, an appealing alternative to vaccination. EBBC’s mode of action remains partially unclear. The results of the presented study have indicated that it may not inhibit the early stages of viral replication. However, its components could affect other aspects of viral entry or infection progression, particularly given the complexity of viral entry mechanisms, including endocytosis (Bayati et al., 2021; Cossart and Helenius, 2014). Despite this, EBBC appears to protect against viral infections or reduce viral exposure to levels manageable by the immune system, potentially inducing the synthesis of virus-specific antibodies tailored to circulating strains.

EBBC offers several features that may be advantageous compared with conventional small-molecule antivirals or experimental biological therapies (**Table 7**). First, EBBC demonstrates broad-spectrum, rapid virucidal activity *in vitro*, achieving ≥4 log10 reductions (≥99.99%) against a diverse panel of both enveloped and non-enveloped viruses at low concentrations (effective concentrations down to 0.01–0.2%), and within short contact times (from 5 minutes), as shown in this study. This potency at low concentrations suggests utility for topical or local prophylactic/therapeutic applications (e.g., surface disinfectants, nasal or ocular formulations) where high local antiviral effect with limited systemic absorption is desirable. Second, the *in vitro* cytotoxicity profile is favorable: EBBC exhibited minimal toxicity at virucidal concentrations in our SRB assays and even stimulated fibroblast proliferation while showing concentration-dependent epithelial effects, indicating a potentially wide therapeutic window for localized use. Third, unlike single-target antivirals, EBBC is a complex mixture that likely acts through multiple concurrent mechanisms (lysozyme and other antimicrobial peptides, organic acids, and marine plasma electrolytes that can disrupt envelopes and viral particles), which reduces the likelihood that a single mutation would confer resistance and may provide robust activity against emerging variants. Finally, EBBC demonstrated stability (retention of virucidal efficacy after 12 months) and is derived from naturally occurring components, which could translate to acceptable tolerability for topical use. That said, there are important caveats: EBBC composition can vary depending on source and requires standardization; bee-product allergic potential (e.g., hypersensitivity to bee proteins) must be carefully evaluated and all current data are *in vitro*. Therefore, definitive claims about comparative safety and systemic use cannot be made without targeted *in vivo* toxicology, pharmacokinetic assessments, and controlled clinical studies. Until such studies are performed, EBBC should be considered a promising topical/adjunct antiviral candidate rather than a replacement for approved systemic antiviral therapies or vaccines.

**Table 7:** Comparative overview of EBBC and antiviral therapies.

Feature	EBBC	Small-molecule antivirals (e.g., oseltamivir, remdesivir)	Monoclonal antibodies	Chemical disinfectants (alcohols, peroxides)
Spectrum of activity	Broad, both enveloped and non-enveloped viruses (*in vitro*)	Typically narrow, virus- or family-specific	Very narrow, strain-specific	Broad, but mostly against enveloped viruses
Speed of action	Rapid virucidal effect within minutes at low concentrations	Slower, require replication inhibition over hours–days	Immediate neutralization after binding	Very rapid (seconds–minutes)
Mechanism	Multifactorial (enzymes, peptides, acids, electrolytes disrupt particles and membranes)	Single molecular targets (polymerase, protease, entry)	Specific binding to viral epitopes	Denaturation of proteins and membranes
Resistance risk	Low (multi-component, multi-target)	High (point mutations can confer resistance)	High (escape mutations)	Low (non-specific physicochemical action)
Potency at low concentrations	Effective at 0.01–0.2% *in vitro*	Active at micromolar range intracellularly	Potent at nanomolar range *in vivo*	Usually require ≥60–70% concentrations
Safety/tolerability	Promising *in vitro* cytotoxicity profile; possible topical use; systemic safety unknown	Variable, systemic adverse effects known	Generally safe, but risk of infusion reactions	Not suitable for internal use; cytotoxic
Formulation/application	Potential topical (nasal, ocular, skin), disinfectant, adjunct	Oral or IV systemic therapy	IV or subcutaneous systemic therapy	Only for environmental/skin surface use
Limitations	Source variability, possible allergenicity, no *in vivo* data yet	Virus-specific, resistance, cost	High cost, cold-chain, strain specificity	Irritant, not usable internally

## 5 Conclusion

Our research shows that EBBC system is both versatile and adaptable, showcasing a unique capacity to combat viruses impacting human health. The innovative inoculation step results in high antiviral activity while minimizing cytotoxicity. This highlights the significance of the technology in addressing both known and emerging viral threats, positioning it for readiness against potential pandemics, including Disease X.

## Data Availability

The raw data supporting the conclusions of this article will be made available by the authors, without undue reservation.
